# Positive Outcomes of Physiotherapy Intervention in a Wedge Compression Fracture of the L1 Vertebra: A Case Report

**DOI:** 10.7759/cureus.51774

**Published:** 2024-01-06

**Authors:** Richa S Gandhi, Shruti S Bhoge, Tejaswini Fating, Rutuja G Sawalkar

**Affiliations:** 1 Community Health Physiotherapy, Ravi Nair Physiotherapy College, Datta Meghe Institute of Higher Education and Research, Wardha, IND

**Keywords:** vertebral compression fracture, road traffic accidents, physiotherapy, exercise, injuries, vertebral, posterior decompression spinal fusion, wedge compression fracture

## Abstract

The thoracolumbar spine is prone to vertebral compression fractures (VCFs). An injury mechanism known as flexion compression is responsible for thoracolumbar spine compression fractures. Usually, this mechanism affects the longitudinal ligament at the front and the front part of the vertebral body as the first components. Pain is the first and foremost symptom; here we present a case report of a 34-year-old male, who came to the hospital with complaints of back pain, and difficulty in breathing followed by a road traffic accident (RTA). MRI and X-ray investigations were done. The patient was diagnosed with a fracture of the anterolateral aspect of the right fourth and fifth ribs and posterolateral aspect of the sixth rib, acute anterior wedge compression fracture of the L1 vertebra, and bilateral minimal pneumothorax and haemothorax. The patient was managed surgically with post-decompression and spinal fusion at the D12-L2 level. The outcomes used were the Oswestry Low-Back Disability Questionnaire, the numerical pain rating scale, and Manual Muscle Testing (MMT). This case report specifies the physiotherapeutic rehabilitation protocol, mainly focusing on techniques like breathing exercises, and upper limb and lower limb strengthening along with trunk and pelvic floor muscles strengthening.

## Introduction

Road Traffic Accidents (RTAs) are still the nation's primary cause of fatalities, injuries, and hospital admissions [1]. RTAs are among the most frequent causes of vertebral fractures in the evolving world. In today's environment, there is a significant risk of suffering multiple damages in car accidents, including major spinal injuries. The most frequent way that spinal fractures occur is from a car flipping over [2]. By definition, a vertebral compression fracture (VCF) compromises the fracture of the anterior segment of the spine, which in turn compromises the anterior longitudinal ligament and the front half of the vertebral column [3]. There are three types of VCF, namely crush, biconcave, and wedge. Over half of all VCFs are wedge fractures, making them the most prevalent type [4]. Along with causing back discomfort and spinal instability, a VCF can result in severe sequelae such as respiratory infections, kyphosis, blood clots in the lower extremities, and even death when proper conservative therapy is not followed [5]. Two surgical techniques that have shown a lot of potential for the correction of spinal compression fractures are kyphoplasty and vertebroplasty [6].

Traffic accidents were also the main source of chest trauma. The most common injuries that were noticed were haemothorax (62.5%), fractures (70.3%), and rib fractures (73.4%) [7]. Rib fractures can puncture the lung parenchyma and lead to pneumothorax. This injury can lead to discomfort, difficulty breathing, and decreased chest expansion [8]. When the acute pain from a fracture subsides, a walking program can start, along with mild strengthening activities that target the spinal extensor muscles. Bracing is frequently utilized in acute nonsurgical therapy. For certain individuals, early mobilization and ambulation can be encouraged and supported by a home physiotherapist [9]. Physiotherapy plays a major role in the management and rehabilitation of spinal compression fractures. Common physiotherapy techniques and methods are breathing techniques, gentle spinal stretches, upper and lower limb mobility, patient education, and core strengthening activities. Physiotherapy is crucial in helping patients with wedge compression fractures return to their regular daily routines [10]. In this report, we are discussing the post-operative rehabilitation of a 34-year-old male patient sustaining an L1 vertebra wedge compression fracture and rib fracture with pneumothorax.

## Case presentation

Patient information

A 34-year-old male was brought to Acharya Vinoba Bhave Rural Hospital casualty by his family with a history of RTA due to the car overturning on September 13, 2023, with complaints of difficulty in breathing, backache, and bony crepitations on the anterior chest wall on the fourth and fifth ribs. The patient had no history of loss of consciousness, and his Glasgow Coma Scale score was found to be normal (E4V5M6). The pain was sudden, progressive, and dull aching. It increased during movement and was relieved on medications. The patient rated her pain 8 on the numerical pain rating scale (NPRS). The patient had undergone certain investigations like X-ray of the spine and chest, and he was diagnosed with an acute anterior wedge compression fracture of L1 with fragment impacting at D12-L2 level and a fracture of the anterolateral aspect of the right fourth and fifth ribs and posterolateral aspect of sixth rib. The patient also had a haemothorax. For haemothorax, an intercoastal drainage was placed. The patient underwent spinal fusion at the D12-L2 level and posterior decompression for the wedge compression fracture a month after the RTA.

Clinical findings

The patient was seen in a supine lying posture with the head end elevated to 30^o^. He was mesomorphic, with a BMI of 20 kg/m^2^. His speech, vision, and hearing were normal. During a neurological evaluation, sensations and reflexes were intact. On the American Spinal Injury Association (ASIA) scale, the grade was found to be E, i.e., motor or sensory functions are normal. On motor examination, the range of motion (ROM) and power were evaluated using manual muscle testing (MMT) and chest excursion was assessed, all of which are mentioned in Table 1.

**Table 1:** Pre-rehabilitation findings of MMT, ROM and chest excursion Score: 8/10 - good (according to Kendall's MMT) The normal value of chest excursion at nipple level is 2-3 cm. MMT: Manual muscle testing; ROM: Range of motion; NA: Not applicable

Outcome measures	Pre-rehabilitation findings
Muscle strength of back extensors	NA
ROM of lumbar extension	0-5^0^
Chest excursion (at nipple level)	1.4 cm

Diagnostic findings

**Figure 1:**
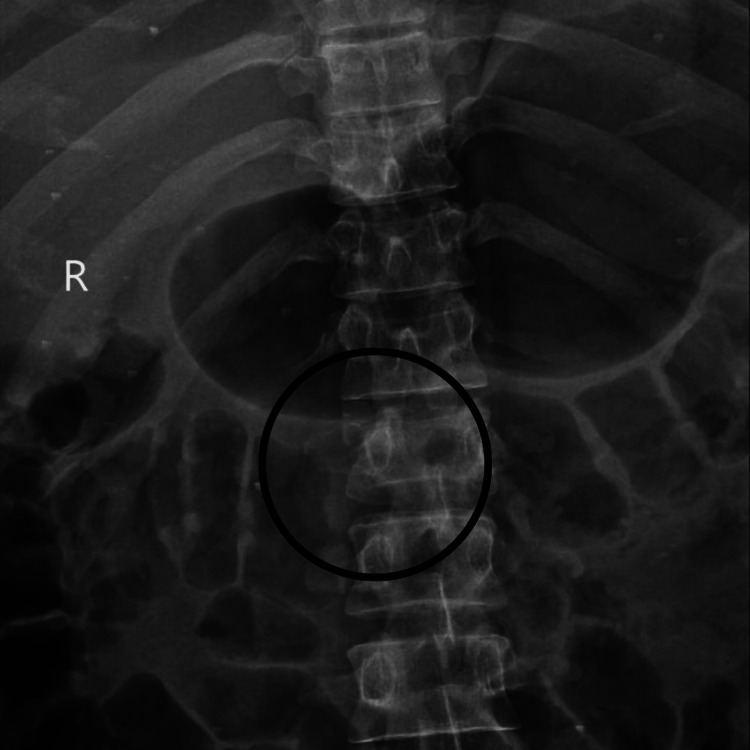
X-ray of spine showing L1 vertebra fracture The circle shows acute wedge compression fracture with retropulsion of the L1 vertebra.

**Figure 2:**
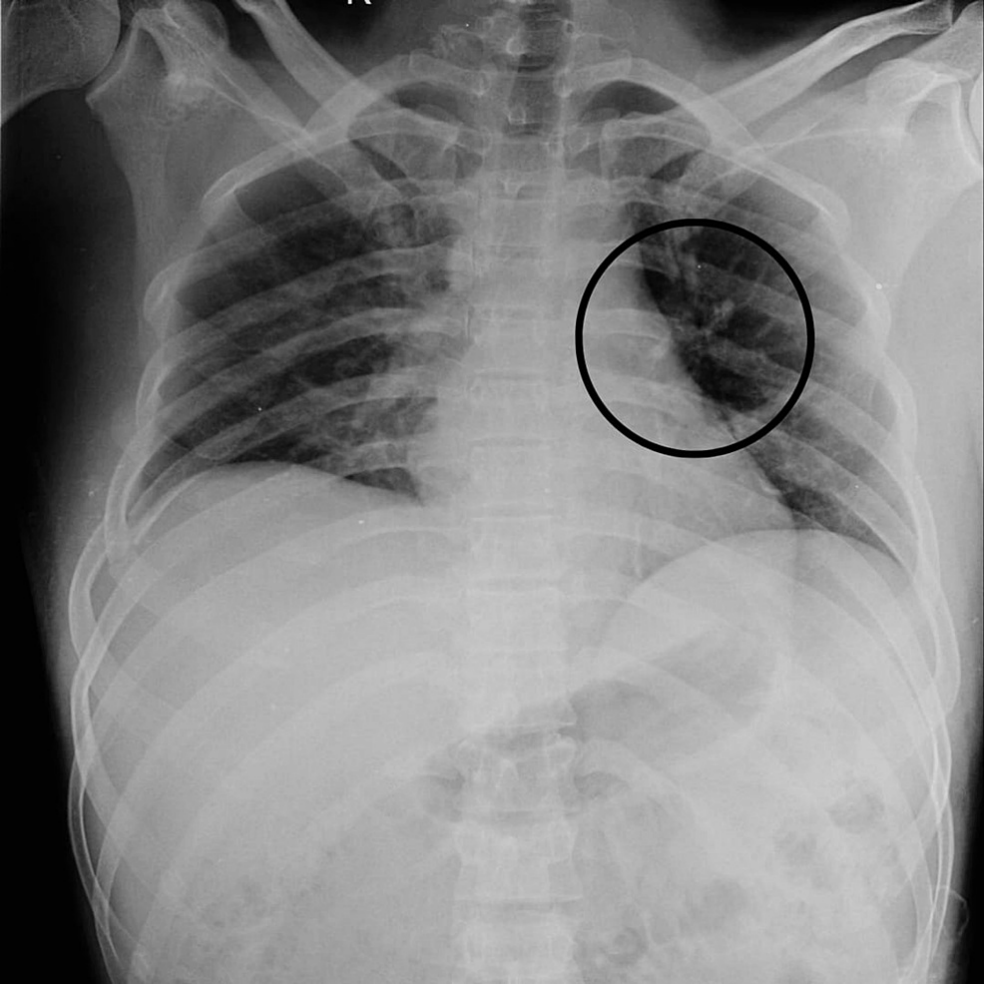
X-ray of chest showing fourth, fifth, and sixth rib fracture The circle shows a fracture of the anterolateral aspect of the right fourth and fifth ribs and the posterolateral aspect of the sixth rib.

**Figure 3:**
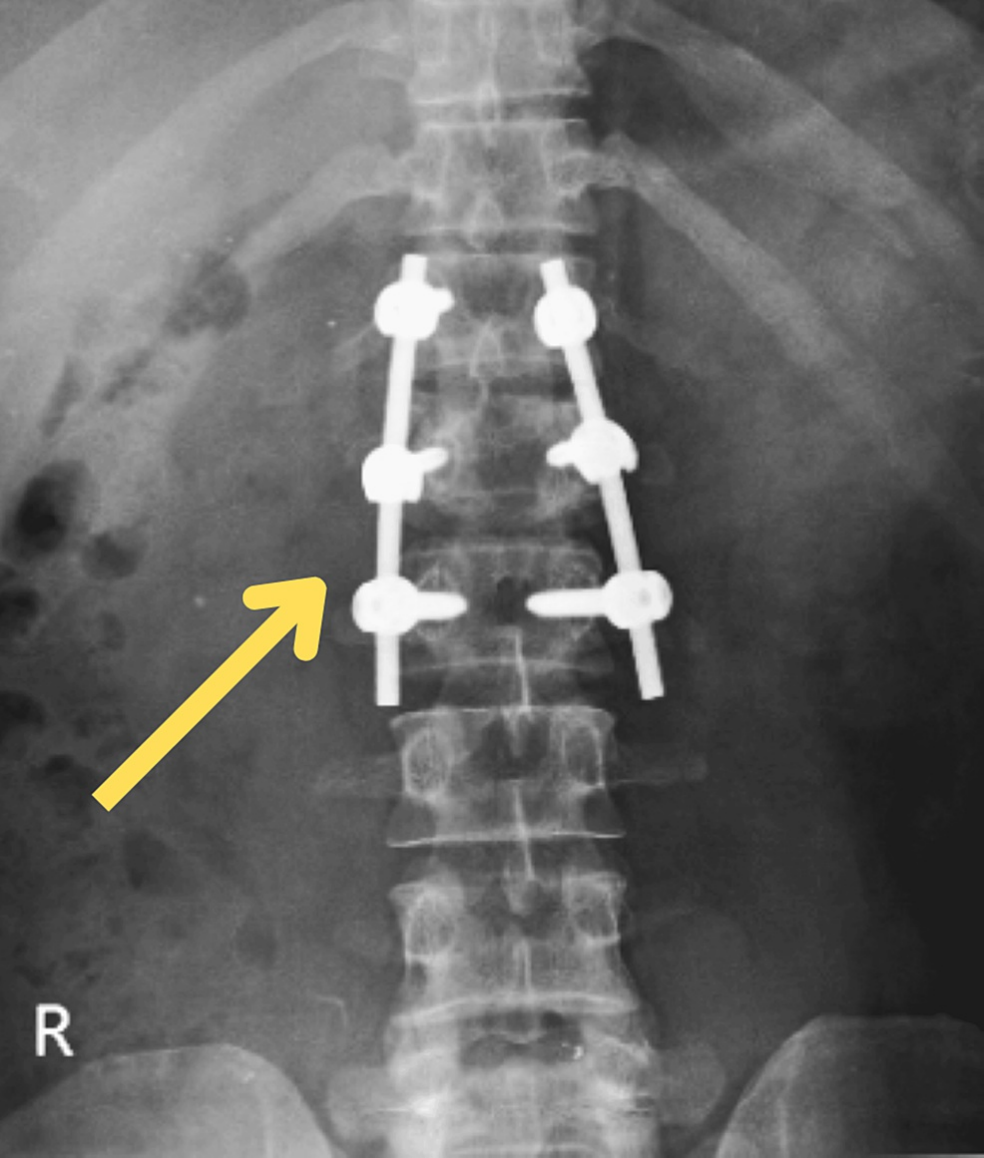
Post-operative X-ray of the spine The yellow arrow points towards the spinal fusion at the D12-L2 level.

An X-ray of the chest and MRI of the dorsal spine were performed as a diagnostic tool, which revealed haemothorax, a fracture of the anterolateral aspect of the right fourth and fifth ribs and posterolateral aspect of the sixth rib, and acute anterior wedge compression fracture of the L1 vertebra involving spinous process with small triangular fragment impacting on anterior dura at D12-L1 level. The above features favour a diagnosis of acute wedge compression fracture with retropulsion of the L1 vertebra involving a spinous process causing canal stenosis at the D12-L1 level and a fracture involving the fourth, fifth, and sixth ribs. Both fractures are illustrated in Figures 1, 2. Post-operative radiograph shows spinal fusion at the D12-L2 level, depicted in Figure 3.

Physiotherapy intervention

**Figure 4:**
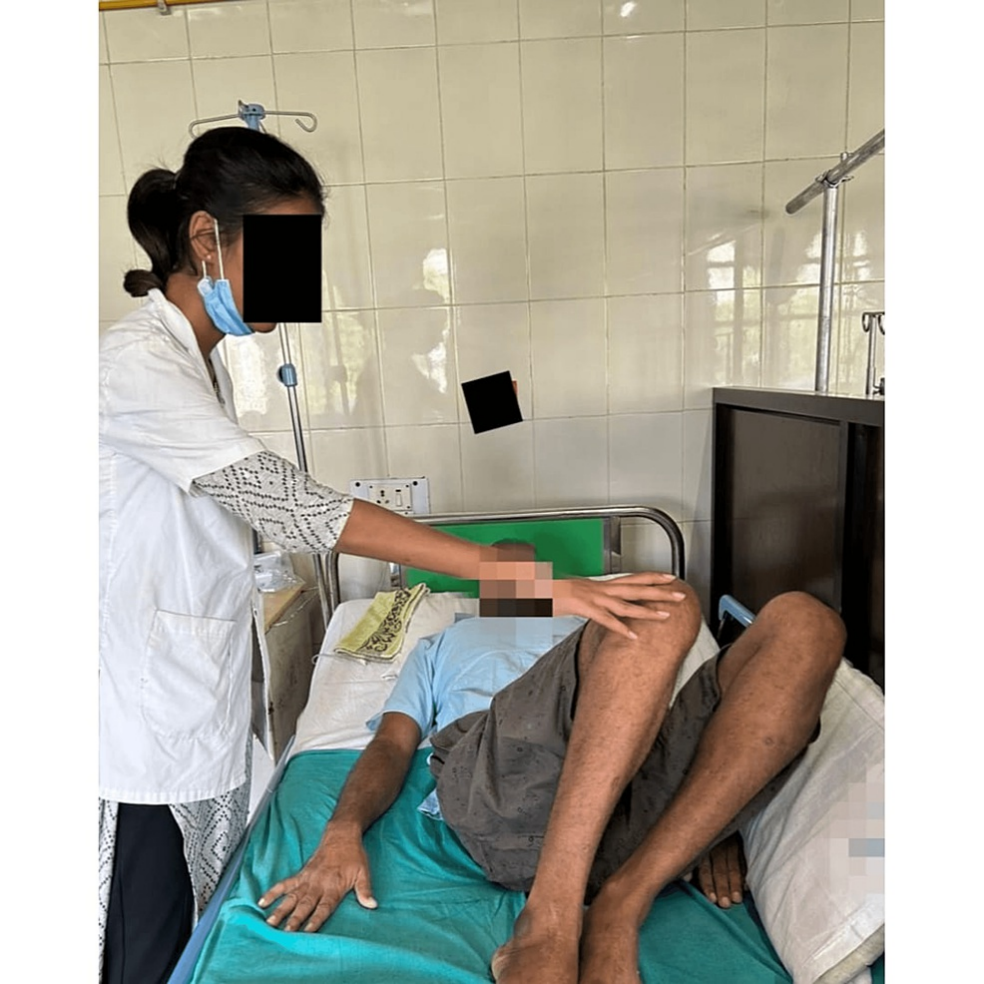
Pelvic bridging

Avoiding secondary complications such as deep vein thrombosis, bed sores, and respiratory issues was the main goal of the patient's care. Training in bed mobility was initiated on postoperative day three. The process of strengthening both upper limbs began with a 1 kg weight cuff. Exercises for alignment, bed mobility, trunk stability, and weight shift were provided. Table 2 shows the physiotherapy protocol, and Figure 4 shows the patient receiving physiotherapy treatment. The patient received treatment for five days a week for two weeks after which the patient was discharged.

**Table 2:** Physiotherapy protocol reps: repetitions; secs: seconds; NA: Not applicable

Problem list	Goals	Physiotherapy intervention	Progression
Difficulty in breathing	To avoid respiratory problems.	Pursed lip breathing and diaphragmatic breathing.	Breathing with 10 secs hold. 10 reps × 1 set.
Mobility issues	To improve bed mobility.	Log rolling, side-lying to sitting bedside, sit to stand.	NA
Secondary complications such as venous thrombosis, bed sores, etc.	To avoid this follow-up issue.	Ankle-toe movement every two hours and altering the position.	10 reps × 1 set of ankle toe movements
The weakness of pelvic floor muscles	To reinforce pelvic floor muscles.	Pelvic floor exercises and bridging unilateral as well as bilateral.	10 secs hold, 10reps × 1 set
Insufficiency in the trunk and core muscles	To build up the core muscles needed for balance and trunk stability.	Back extension exercise, quadripod, reach outs.	10 reps × 1 set
Early fatigue and difficulty in coughing	To improve lung volume capacity.	Thoracic expansion exercises, spirometry and teaching coughing mechanism.	10 reps × 1 set
The patient's and the family's education	Encourages and keeps up a patient's positive attitude on their course of therapy, hence promoting a quicker recovery.	The individual receiving treatment and family members were provided with thorough explanations regarding their medical situation and the importance of physical therapy activity.	The home program was explained.

Follow-up and outcome measures

After treatment outcome measures like the NPRS, MMT, ROM, and the Oswestry Low Back Disability Questionnaire were evaluated. The findings of the outcome measure are shown in Table 3.

**Table 3:** Outcome measures NPRS: Numerical pain rating scale; MMT: Manual muscle testing; ROM: Range of motion; NA: Not applicable

Sr no.	Scales	Pre-rehabilitation	Post-rehabilitation
1.	NPRS	8/10	3/10
2.	Oswestry Low Back Disability Questionnaire	36/50	25/50
3.	MMT Of back extensors	NA	4/10
4.	ROM Of lumber extension	0^o^-5^o^	0^o^-16^o^
5.	Chest excursion	1.4 cm	2.6 cm

## Discussion

RTAs are a major and growing global factor in hospitalization, bodily harm, and fatality rates [11]. Globally, the most common aetiology for spinal cord injury remains RTA, followed by falls [12]. VCFs are linked to significant morbidity, such as elevated mortality, diminished lung capacity, and a decreased standard of life. Kyphoplasty and spinal surgery, two relatively new components to the treatment, provide spinal enlargement [13]. Patients with mild to severe lower vertebra fractures that are compression-related may benefit from a mix of conventional rehabilitation techniques [14]. Patients with chronic vertebral column injury require an enduring, strong rehabilitation program. Active avoidance and management of potential issues are necessary to lower the rate of mortality from complications [15].

In our study, we started back extensor strengthening for the patient, which led to improved strength of back extensor muscles and back extension range. In their study, Bergström et al. showed improved back extensor strength after a five-week strengthening program in vertebral fracture cases in postmenopausal women [16]. Sum et al. conducted a study on patients with traumatic ribs and gave incentive spirometry to prevent secondary complications and improve lung volume and capacities, finding improved outcomes [17]. We included spirometry and thoracic expansion exercises in our protocol, and we found improved chest excursion in the patient.

Rehabilitation protocol should include weight-bearing mat exercise orthosis for ambulation and home-based workout routines, which should all be part of the therapy protocol, along with a vigorous strengthening program for the upper limb; techniques for breathing were offered to increase pulmonary compliance [18]. For lower limbs, stretching was provided to avoid muscle tightness and exercises for transferring between beds were taught to prevent bed sores and other issues. The patient and their family members were also introduced to positioning at regular intervals. To enhance sitting stability in both passive and active scenarios, workouts focused on stabilizing the trunk were included. To assist with wheelchair transfer in the years to come, scooting motions and pelvic adjustments were also instructed [19]. Early rehabilitation involvement by the patient helped him avoid more significant complications including joint contractures and pressure sores. Early rehabilitation assistance is essential to the healing of patients with spinal column injuries [20].

## Conclusions

The results of this case study highlight how crucial physical therapy exercises and interventions are in helping patients perform daily living activities. According to the results of this study, the patient's early physiotherapy intervention helped to maintain appropriate chest compliance and enhance trunk control, as well as prevent serious postoperative problems. Early physical therapy intervention is critical to the healing of individuals with fracture injuries, and it should begin as soon as feasible.
